# Robust, Precise, and Deep Proteome Profiling Using a Small Mass Range and Narrow Window Data-Independent-Acquisition Scheme

**DOI:** 10.1021/acs.jproteome.3c00736

**Published:** 2024-01-26

**Authors:** Klemens Fröhlich, Regula Furrer, Christian Schori, Christoph Handschin, Alexander Schmidt

**Affiliations:** † Proteomics Core Facility, Biozentrum Basel, University of Basel, 4056 Basel, Switzerland; ‡ Biozentrum Basel, University of Basel, 4056 Basel, Switzerland

**Keywords:** proteomics, DIA, narrow mass range, deep proteome profiling, robust quantitation, WikiPathWay enrichments, murine skeletal muscle, PGC-1α

## Abstract

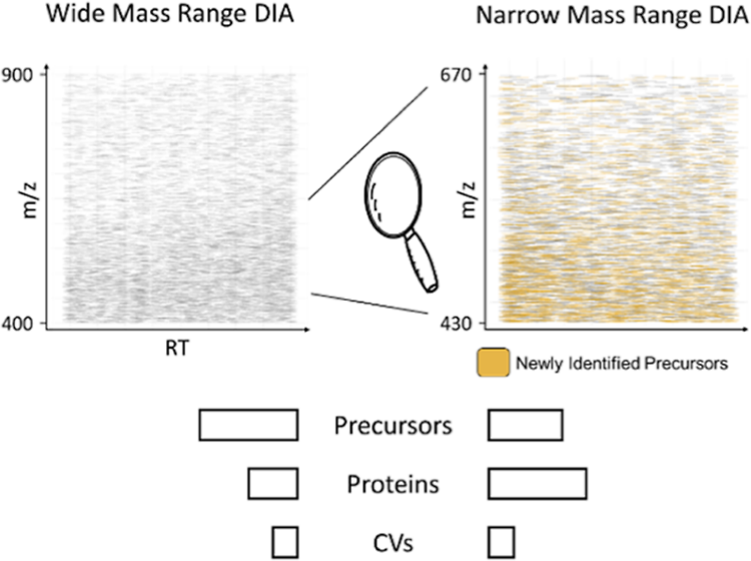

In recent years, a plethora of different data-independent acquisition methods have been developed for proteomics to cover a wide range of requirements. Current deep proteome profiling methods rely on fractionations, elaborate chromatography, and mass spectrometry setups or display suboptimal quantitative precision. We set out to develop an easy-to-use one shot DIA method that achieves high quantitative precision and high proteome coverage. We achieve this by focusing on a small mass range of 430–670 *m*/*z* using small isolation windows without overlap. With this new method, we were able to quantify >9200 protein groups in HEK lysates with an average coefficient of variance of 3.2%. To demonstrate the power of our newly developed narrow mass range method, we applied it to investigate the effect of PGC-1α knockout on the skeletal muscle proteome in mice. Compared to a standard data-dependent acquisition method, we could double proteome coverage and, most importantly, achieve a significantly higher quantitative precision, as compared to a previously proposed DIA method. We believe that our method will be especially helpful in quantifying low abundant proteins in samples with a high dynamic range. All raw and result files are available at massive.ucsd.edu (MSV000092186).

## Introduction

The field of proteomics has seen a variety of novel data-independent acquisition (DIA) methods that aim at overcoming the stochastic nature of data-dependent acquisition (DDA) methods by analyzing predefined mass windows in either MS1 or MS2 mode. This resulted in consistent and reproducible data acquisition across samples and facilitated their quantitative comparison. However, compared with DDA, it also required the use of much wider mass isolation windows to fragment all precursor ions within a mass spectrometer (MS) cycle. Consequently, the generated tandem mass spectra were highly multiplexed and were more challenging to analyze.

Historically, the first viable DIA approach was first introduced using a Fourier-transform ion cyclotron resonance (FT-ICR) MS^1^ and has seen a variety of developments over the years, as reviewed in Chapman et al.^2^ For example, Purvine et al. introduced Shotgun CID, which was implemented on a time of flight MS and used in-source fragmentation to analyze all eluting precursor ions in an MS cycle.^3^ The method was modified by Waters to allow all ion fragmentation in a collision cell and is referred to as MS^E^.^4^ Another pioneering approach was introduced by Venable et al., introducing DIA on an ion trap MS device by applying large mass isolation windows for MS/MS analysis that cover the entire precursor mass range during one MS cycle.^5^ All these and other methods inspired later implementations of DIA such as Precursor Acquisition Independent From Ion Count (PAcIFIC) and Sequential Window Acquisition of all Theoretical Mass Spectra (SWATH-MS).^6,7^

Today, DIA methods have gained momentum for quantitative bottom-up proteomics. It has been shown that DIA can outperform DDA in terms of proteome coverage and quantitative performance.^8,9^ This also holds true for many specialized applications, such as glycoproteomics or phosphoproteomics.^10,11^ Nonetheless, it is important to highlight that DDA remains a highly effective tool for many applications that require high-quality tandem mass spectra with a low degree of interferences. For instance, it offers the ability to perform open searches, an invaluable tool for the discovery of unknown post-translational modifications.^12^

Many different flavors of DIA are available due to the possible intricate combinations between chromatographic setup, tandem MS (and its settings), and the analysis software being used. This is an extremely active field of research with new methods and software constantly being developed. It also includes methods for general or more specialized applications, ranging from ultrafast chromatography^13^ to ultrasensitive applications^14^ to near-complete coverage of an entire proteome. While the latter is feasible by using orthogonal sample preparation methods and multiple measurements of prefractionated samples, this method is time-consuming and therefore not well suited to quantitatively compare a high number of samples. The first near comprehensive single-shot DIA analysis capturing more than 10,000 proteins in a single run was performed by Muntel et al.,^8^ showcasing the potential for deep and single-shot DIA analysis. For reference, Bekker-Jensen et al. estimated that the HeLa proteome consists of ∼12,200 proteins, indicating that around 82% of the expected proteins were identified in the single-shot DIA analysis.^15^ However, the extremely long gradients and extensive library generation measurements required in this setup considerably limit the sample throughput capabilities of this approach.

Recently, new data analysis tools have been introduced that allow for library-free searches of DIA data and thereby alleviate the need for time-consuming and potentially costly spectral library generation measurements. This immensely improved the ease of use of DIA and allows for the more widespread use of DIA in quantitative proteomics. Using this new library-free DIA analysis software tools, Kawashima et al. demonstrated a 2 h gradient method capable of identifying more than 10,000 proteins in a HEK lysate.^16^ This was achieved by focusing on a smaller mass range (500–740 *m*/*z*) in combination with a high field asymmetric waveform ion mobility spectrometry (FAIMS) device. Furthermore, a staggered window placement approach was used, which can increase proteome coverage.^17^ In brief, the isolation windows for peptide fragmentation are not constant between fragmentation cycles but are offset by usually 50% of the window width. The fragment spectra can later be demultiplexed, resulting in fragment spectra with reduced complexity due to their in silico generated smaller isolation window width.^18^ While an impressive proteome coverage was achieved with this approach, a thorough evaluation of its quantitative performance is still missing.

In this study, we set out to investigate the quantitative accuracy and precision of deep proteome analyses using a narrow mass range with regard to our needs as a proteomics core facility. As we want to provide users with the most reliable information possible, we adapted the recent method published by Kawashima et al. to achieve higher quantitative precision as well as a simplified mass spectrometry setup and more robust liquid chromatography (LC) parameters, while still achieving over 9300 protein identifications using only 150 ng of peptides and a 2 h gradient.

We then applied our newly developed method to dissect the proteome profile of mice lacking the peroxisome proliferator-activated receptor γ coactivator 1α (PGC-1α) specifically in skeletal muscle.

Compared to a standard DDA method, we could double proteome coverage and, most importantly, achieve a significantly higher quantitative precision as compared to the originally proposed narrow mass range DIA method by Kawashima et al.

## Materials and Methods

### Proteomics Sample Preparation for Benchmark Analyses

Cell lysates of either human HEK293T or *E. coli* K12 were heated and reduced at 95 °C for 10 min (lysis buffer contained 5% SDS, 10 mM TCEP, and 100 mM triethylammonium bicarbonate). Proteins were alkylated using 15 mM iodoacetamide at 25 °C in the dark for 30 min. For each sample, 50 μg of protein lysate was captured, digested (trypsin 1/50, w/w; Promega), and desalted using S-Trap cartridges (Protifi) following the manufacturer’s instructions. Resulting peptides were dried and stored at −20 °C until measurement. Samples were then resuspended at a final concentration of 200 ng/μL.

### Experimental Animals

To generate muscle-specific PGC-1α knockout mice (KO), we crossed PGC-1α^flox/flox^ C57BL/6 mice with a HSA-cre mouse line (Jackson Laboratories stock number: 009666), as described previously.^19,20^ The floxed littermates served as wild-type (WT) controls. Mice were housed under standard conditions with a 12 h light/12 h dark cycle and had free access to a regular rodent chow diet. For this study, we used male mice at the age of 18–20 weeks. The experiments were approved by the Kantonales Veterinäramt Basel-Stadt and followed Swiss guidelines for animal experimentation and care.

### Sample Preparation of Murine Specimens

Quadriceps muscles were removed and snap-frozen in liquid nitrogen and stored at −80 °C. After pulverizing the muscle using an ice cold metal mortar, 10 mg was resuspended in lysis buffer (5% SDS, 10 mM TCEP, and 0.1 M TEAB) and lysed by sonication using a PIXUL multi-sample sonicator (Active Motif, CA, USA) with pulse set to 50, PRF to 1, process time to 20 min, and burst rate to 20 Hz. Lysates were incubated for 10 min at 95 °C, alkylated in 20 mM iodoacetamide for 30 min at 25 °C, and proteins digested and purified using S-TrapTM micro spin columns (Protifi, NY, USA) according to the manufacturer’s instructions.

### LC–MS Measurement

All measurements were performed on an Orbitrap Exploris 480 system coupled to a Vanquish NEO system (both Thermo Fisher Scientific, MA, USA). Columns with an ID of 75 μm were self-packed, as described before with C18-AQ 1.9 μm Dr. Maisch beads to a length of 30 cm.^21,22^ Column temperature was kept constant at 60 °C. 120 min gradients were used for peptide separation. Buffer A consisted of 0.1% formic acid, and buffer B consisted of 0.1% formic acid in 80% acetonitrile. For the standard mass range measurements, which we used as a DIA control method (referred to as standard DIA method) (400 to 900 *m*/*z*), the following gradient was employed at 200 nL/min: 0 min: 4% B, 10 min: 10% B, 100 min: 35% B, and 120 min: 50% B. For the narrow mass range measurements, the following gradient was employed at 200 nL/min: 0 min: 4% B, 12 min: 7% B, 108 min: 30% B, and 120 min: 45% B.

For all narrow window acquisition methods, an isolation window of 4 *m*/*z* was used. For the standard mass range acquisition methods, an isolation width of 8 *m*/*z* was used with 1 *m*/*z* overlap always covering 400 to 900 *m*/*z* (referred to as the “standard DIA method”). Staggering refers to a window placement with a 50% overlap. Ion injection time was set to 22 and 55 ms when employing an MS2 resolution of 15,000 and 30,000 at 200 *m*/*z*, respectively.

The initial narrow window acquisition method covered 500–740 *m*/*z* with 4 *m*/*z* staggered windows at 30,000 resolution (referred to as modified Kawashima method). The final optimized method consisted of a survey scan with a resolution of 60,000 at 200 *m*/*z* and MS2 scans with a resolution of 30,000 at 200 *m*/*z*. The optimized DIA scheme covered a range between 430 and 670 *m*/*z* with 4 *m*/*z* isolation windows without staggering and without overlap using optimized window placement. All LC and MS parameters are also available via the deposited MS raw files (massive.ucsd.edu MSV000092186).

### Data Analysis

In total, three different software suites were used. Fragpipe (version 19 employing DIA-NN 1.8.2 beta) was used with the workflow “DIA_Speclib_Quant” and “LFQ-MBR” for DIA and DDA analyses, respectively. Spectronaut (version 17) was used with standard settings in the “DirectDIA+” mode. DIA-NN (version 1.8.1) was used with heuristic protein inference on the protein names level. Neural networks were used with single-pass mode, MBR, and the quantification strategy was set to robust LC (high precision). Databases were sourced from UniProt in November 2021, wherein only reviewed entries for human and *E. coli* proteins were allowed. For the murine database, one entry per gene was obtained from Uniprot (Nov 2021).

The deep proteome profiling data set of murine muscle was obtained from ProteomeXchange via the identifier: PXD000288.^23^ The raw data were downloaded and reanalyzed with FragPipe, as described above. For the reference mass range distribution of precursors in DIA, the data set from Muntel et al. was obtained from ProteomeXchange via the identifier: PXD011691.^24^ The raw data were reanalyzed with DIA-NN, as described above. The data from Kawashima et al. were obtained from ProteomeXchange via the identifiers: PXD029853 and PXD029853 and reanalyzed using DIA-NN, as described above.

Downstream analysis was performed using R with the following packages: tidyverse,^25^ RColorBrewer,^26^ Peptides,^27^ and limma.^28^ The coefficient of variance of precursors was calculated using the “EG. TotalQuantity..Settings.” of the Spectronaut output and “Precursor.Quantity” of the DIA-NN outputs. For protein quantities, the “PG.Quantity” columns of all outputs were used.

Boxplots show median (center line), interquartile range (IQR) where the lower and upper hinges correspond to the first and third quartiles (the 25th and 75th percentiles), and 1.5 × IQR (whiskers). Outliers are not depicted.

## Results and Discussion

### Setup of Narrow Mass Range DIA Methods

DIA has been increasingly used over the past few years in quantitative proteomics, and new methods are constantly being developed to address a wide range of applications. However, there are hardware limitations, and one needs to consider: instruments possess a finite acquisition speed, which means that the time for each measurement cycle (“cycle time”) is crucial. The cycle time influences how many data points can be obtained as a peptide ion is analyzed. When broader isolation windows are used, more peptide ions are cofragmented, resulting in the more complex fragment spectra and limiting the dynamic range. In essence, scientists must balance three key factors: (I) cycle time, (II) covered mass range, and (III) the size of the isolation window. Historically, gas phase fractionations were employed to alleviate slow acquisition speed, meaning that the same sample was injected repeatedly, each time focusing on a different narrow mass range.^6,29,30^ Recently, it was shown that focusing on narrow mass ranges can benefit protein coverage.^31,32^ The rationale can be compared to using a magnifying glass on a painting: while the overall picture is not represented, it is possible to observe intricate details that might be overlooked at a broader glance. As we do not need to detect and quantify every peptide of a protein for its quantitation, it is sufficient to capture a smaller mass range to quantify less precursors overall, which in turn represent more proteins. The approach by Kawashima et al., which focused on a narrow mass range combined with a FAIMS device and the loading of relatively large sample amounts (>1000 ng), achieved nearly comprehensive proteome coverage (∼10,000 proteins) in just 2 h of measurement time, eliminating the need for extensive library building from empirical data.^16^

To evaluate if this method can be implemented into a core facility setup for routine global discovery proteomics analysis, we systematically assessed all relevant MS settings regarding identification rates and quantitative accuracy and precision using an artificial two-protein benchmark sample. The aim of this evaluation was to simplify this promising DIA method as much as possible to reduce possible sources of variation and make it easy to use and robust without compromising on performance.

We assessed whether the suggested high sample amounts used per LC–MS analysis are really required as this might lead to peak tailing, reduced column lifetime and might not be available. Kawashima et al. observed an improvement in proteome coverage with FAIMS only when loading >1000 ng of sample onto their column setup. This is probably related to the fact that using a FAIMS device leads to a drop in signal intensity.^33^ We usually only load 125–250 ng of peptides onto our nano LC setup, to increase robustness. We therefore hypothesized that it would be preferable to eliminate the additional complexity of a FAIMS device, which might also not be available to every MS laboratory and stay in the optimal sample loading range of the nanoLC setup. Consequently, we adjusted this DIA method for LC–MS analysis without using FAIMS and only injecting around 150 ng of sample material (adapted Kawashima method) using the same MS system (Exploris 480). For the first evaluation, we prepared three two-species spike-in samples, which are often used in proteomics,^34,35^ consisting of HEK-293 and *E. coli* K12 in three different concentrations: Only HEK, *E. coli* to HEK 1:20 and *E. coli* to HEK 1:10.

### Assessment of Narrow Mass Range DIA Methods

**Figure 1:**
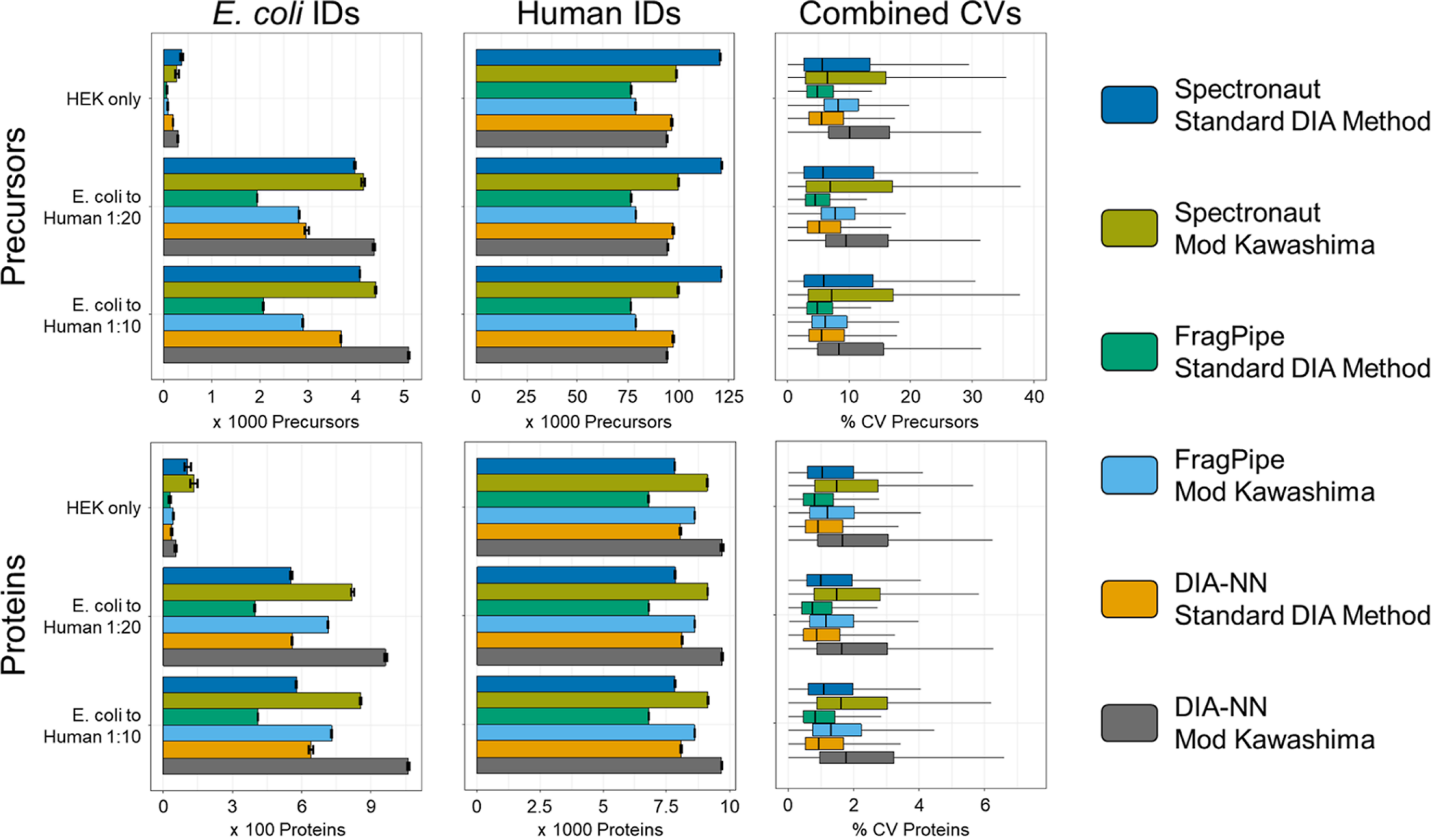
Identification and quantitative variance of measured *E. coli*to HEK spike-in samples. An *E. coli*–HEK dilution series (no *E. coli* = HEK only) was measured in triplicates. Three software tools were used to evaluate two different acquisition methods: Spectronaut version 17, FragPipe version 19.0, DIA-NN standalone version 1.8.1, and standard DIA method = 8 *m*/*z* windows at 15,000 resolution covering 400–900 *m*/*z*. Mod Kawashima = 4 *m*/*z* windows with 2 *m*/*z* overlap (= staggered) at 30,000 resolution covering 500–740 *m*/*z* without using FAIMS. Identifications and variance are divided on precursor and protein level, and identifications are additionally divided into species. The barplots show the mean identifications ± the respective standard deviation of identifications within the group. All displayed identification numbers can be found in Supporting Information Tables 1 and 2.

Before assessing and optimizing the different MS settings, we first tested the capability of different software tools to analyze our quantitative proteomics DIA data acquired over a narrow mass range. We assessed false discovery rates (FDRs) and quantitative accuracy and precision as compared to our standard DIA method, which covers 400–900 *m*/*z* with 8 *m*/*z* windows at 15,000 resolution at 200 *m*/*z* and a 1 *m*/*z* overlapping window placement. We adapted this method from Pino et al., who proposed to use a mass range of 400–1000 *m*/*z*. However, as we lean toward higher quantitative precision, we chose to further decrease cycle time by only targeting a mass range between 400 and 900 *m*/*z*, which only leads to minimally lower IDs.^36^Figure 1 shows the identification of *E. coli* and human precursors and proteins and coefficients of variation (CV) obtained for all different combinations of software tools and methods. We could confirm the observations from the original Kawashima method: While precursor identifications were sacrificed when investigating a smaller mass range, the number of observed protein groups increased for all software suites. Moreover, the number of identified *E. coli* proteins in samples only containing human proteins allowed us to estimate FDR rates for the different methods and software tools. Interestingly, despite the lower number of precursors, the Narrow Window Acquisition Scheme overall seemed to have an FDR comparable to that of our standard acquisition method across all data analysis schemes. However, we observed strong differences in identification FDR for the different data analysis platforms employed. Spectronaut found on average 120,598 precursors in HEK only samples while identifying 377 *E. coli* precursors in the same HEK only samples (FDR of 0.31%) with our standard method and 98,892 human and 267 *E. coli* precursors with the adapted Kawashima method (0.27%). In HEK-only samples, FragPipe found on average 76,721 human and 68 *E. coli* precursors with our standard method (0.09%) and 78,713 human and 88 *E. coli* precursors with the modified Kawashima method (0.11%). In HEK-only samples, DIA-NN found on average 96,699 human and 185 *E. coli* precursors with our standard method (0.19%) and 94,375 human and 288 *E. coli* precursors with the adapted Kawashima method (0.31%). On the protein level, Spectronaut exhibited the highest FDR.

Please note that, in this study, the search space and the presence of human and *E. coli* proteins is not of equal size, and therefore, the FDR estimation used here does not approximate the real FDR achieved by the software suites. Nonetheless, the estimated FDR here does allow for comparisons between the two acquisition methods and the different software suites.

Interestingly, Spectronaut identified by far the most precursors in HEK only samples (120,598 ± 161) suggesting the highest sensitivity of all software tools. However, this was not reflected in the *E. coli* identifications. Here, DIA-NN consistently identified most *E. coli* proteins independent of which acquisition method was used. As *E. coli* proteins were spiked in low concentrations, this suggests that DIA-NN is superior in the identification of lowly abundant proteins, while Spectronaut seems to provide a better coverage (higher ratio of precursors/proteins) of higher abundant proteins.

We also assessed the quantitative accuracy of the modified Kawashima method as compared to our standard mass range DIA method (Supporting Information Figure S1). Interestingly, DIA-NN and FragPipe generally seemed to gain quantitative accuracy when using the adapted Kawashima method, while Spectronaut seemed to lose quantitative accuracy on the precursor level but not on the protein level.

Most importantly, we noticed that the CVs for the adapted Kawashima method were higher than our standard method irrespective of protein or precursor level or which DIA analysis software was used (Figure 1, right column).

Precision is an important metric in differential quantitative proteomics, as higher precision generally leads to higher statistical confidence, even if, like in isobaric labeling approaches, the ratio is slightly distorted.^37−39^ As DIA-NN provided the best trade-off between sensitivity, accuracy, and precision for our modified Kawashima method, we chose to use this software suite for all following DIA MS analyses.

### Optimization of the Quantitative Performance

Next, we investigated whether our implementation led to the observed higher quantitative variance or whether the original Kawashima method itself also suffers from a higher quantitative variance. Therefore, we obtained the raw data of the original Kawashima et al. publication and performed a DIA-NN + R analysis with the exact same parameters used in our own comparison of the modified Kawashima method. As can be seen in Supporting Information Figure S2, the distribution of CVs in the original Kawashima raw data was considerably higher than both our standard and our modified Kawashima methods, suggesting that our implementation already improved quantitative precision over the original Kawashima method.

We then set out to identify the source of the higher quantitative variance of our modified Kawashima method as compared to that of our standard DIA method and, if possible, implement a solution. We had previously conducted measurements in our own laboratory to compare staggered and nonstaggered methods in terms of identification and were only able to identify a minute amount of additional IDs when using staggered methods (data not shown). We hypothesized that while in this setting staggering of DIA windows might lead to a slight increase in IDs, it might also lead to an increased variance in quantitation. Additionally, we noticed that Kawashima et al. found a mass range of 500–740 *m*/*z* leading to a higher proteome coverage as compared to 400–640 *m*/*z* with a FAIMS device. We obtained raw files from a variable windows DIA acquisition from Muntel et al.^24^ and summarized the mass range distribution of our own standard acquisition method (Supporting Information Figure S3). When not using a FAIMS device, we estimated that the mass range 430–650 *m*/*z* should give a coverage similar to that of 500–740 *m*/*z*. It would also allow for slightly quicker cycle times, which again should help increase the quantitative precision by providing more data points per peak. Thus, we repeated the measurement of our dilution series, comparing nonstaggered vs staggered approaches for the mass range suggested by Kawashima et al. (with FAIMS) from 500 to 740 *m*/*z* and for our estimated optimal range (without FAIMS) of 430–650 *m*/*z*.

**Figure 2:**
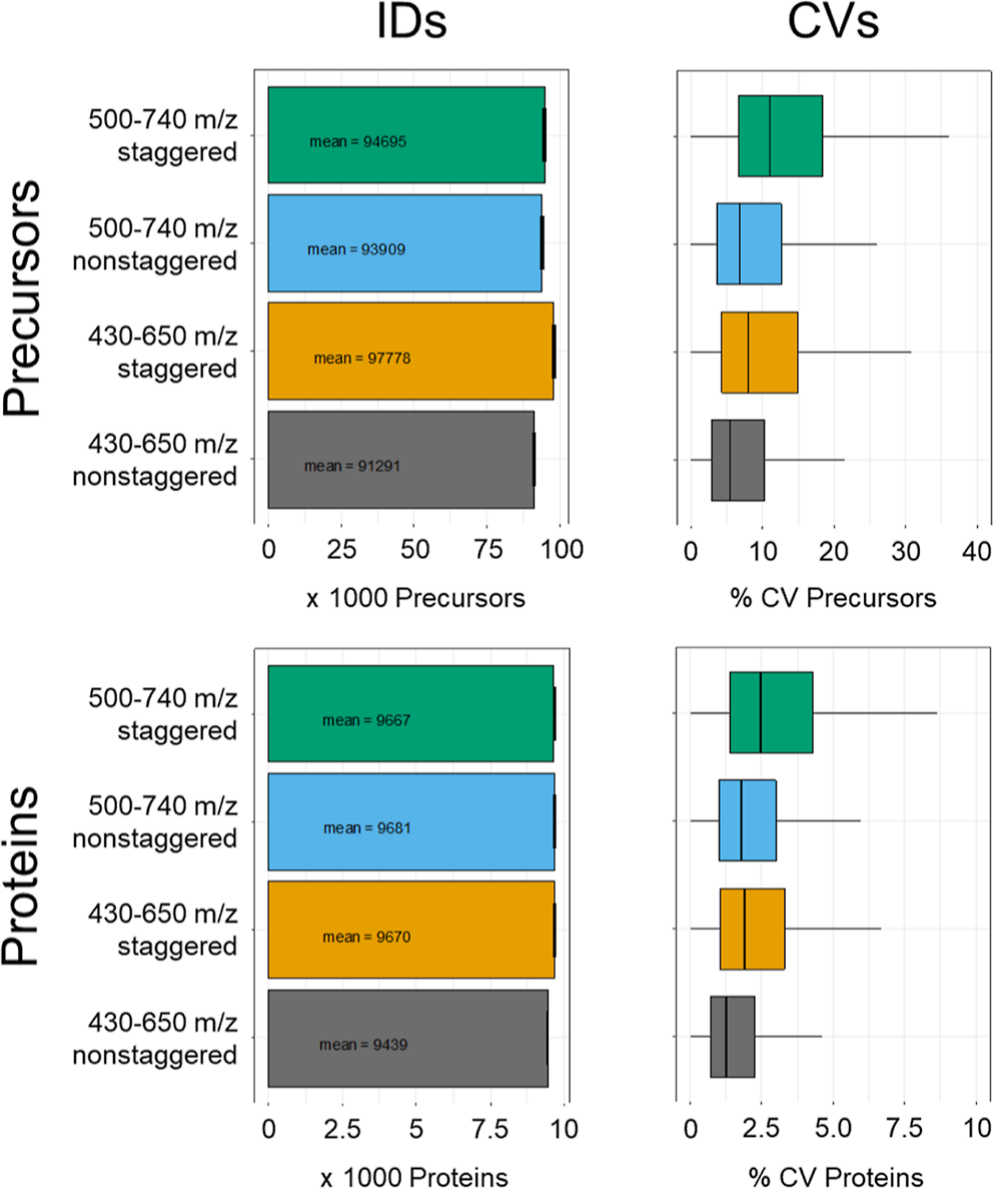
Comparison of identifications and quantitative variance between different mass ranges and isolation window placement schemes.

As shown in Figure 2, without FAIMS, using a mass range of 430–650 *m*/*z* provided similar numbers of precursor and protein identifications as a mass range of 500–740 *m*/*z*. Presumably due to the faster cycle time and resulting higher number of data points, we observed a decrease in CVs when measuring HEK lysates in triplicates. More strikingly, using a nonstaggered DIA method led to a drastic decrease of CVs for both mass ranges on both precursor and protein level. As we always want to provide the most precise quantitation, we chose the 430–650 *m*/*z* mass range without staggering for further experiments. On the protein level, we thereby accepted a drop of proteome coverage from 9670 proteins to 9439 corresponding to ∼2% of protein identifications.

Another interesting feature of the published Kawashima method is the resolution of 45,000 at 200 *m*/*z* at the MS2 level, which allows for 55 ms of “free” injection time suggesting a high degree of sensitivity due to the high injection time as well as a better signal-to-noise ratio for individual fragment ions due to the high resolution. This comes at a high price: As compared to our standard method with a resolution of 15,000, the cycle time is tripled. Consequently, the number of data points across peaks is reduced to a third when applying the same number of isolation windows.

Two mass ranges and two window placement designs were investigated for proteome coverage and quantitative variance by measuring HEK triplicates. A staggered method refers to an isolation window overlap of 50%. All data were analyzed using DIA-NN with a heuristic protein inference set to protein level summary.

We therefore asked if the high resolution of 30,000 and the longer fill times for fragment spectra are really needed for higher proteome coverage. Therefore, we analyzed HEK samples with injection times ranging from 10 to 60 ms in 10 ms increments at both 15,000 and 30,000 MS2 resolution at 200 *m*/*z*. As can be seen in Supporting Information Figure S4, higher injection times and higher resolution led to more identifications, respectively. Interestingly, even at an extremely aggressive maximum injection time of only 10 ms, nearly 50,000 precursors could be identified in a single run when using an MS2 resolution of 30,000 but only around 40,000 precursors were identified when using 10 ms injection time and a resolution setting of 15,000. This suggested that even at 22 ms of injection time (which is “free” injection time at 15,000 resolution), the resolution seemed to be a limiting factor for identifications in DIA-type data analysis. This is especially interesting in the light of recent developments, where higher resolutions from short transients are derived from existing Fourier-transform based MSs by using computationally intensive algorithms.^40,41^

### Comparison of Optimized DIA Acquisition Methods

**Figure 3:**
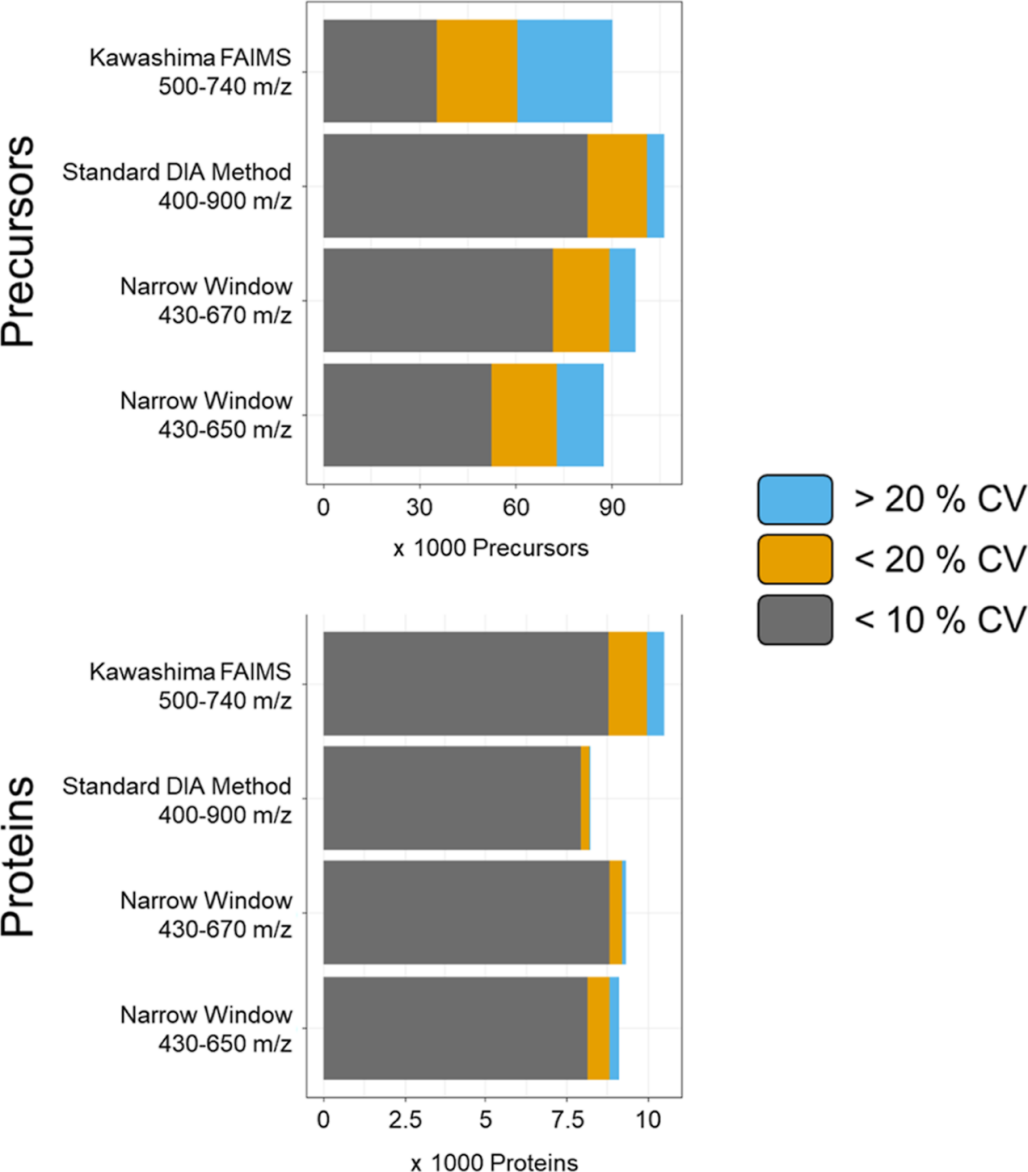
Optimized mass range measurement of HEK triplicates. HEK peptides were injected in triplicate to assess identifications and quantitative variance. Additionally, original data from the publication of Kawashima et al. were reanalyzed by our DIA-NN + R pipeline in order to directly compare quantitative variances.^16^

To evaluate if our DIA approach using this optimized mass range also performs well compared to different acquisition strategies, we analyzed HEK lysates in triplicates, using our (I) standard DIA method, (II) as well as the aforementioned 430–650 *m*/*z* nonstaggered method and (III) additionally a nonstaggered method covering a slightly larger mass range of 240 *m*/*z* (same as the original Kawashima method) from 430 to 670 *m*/*z* to improve proteome coverage. We also visualized the results of our reanalysis of the original Kawashima measurements in this comparison (Figure 3). As expected, the highest number of proteins was observed in the original Kawashima publication. However, especially at the precursor level, only a small fraction of the compounds was quantified with high precision. Conversely, when covering a narrow mass range of 430–670 *m*/*z* and using nonstaggered acquisition windows, we observed similar CV distributions as compared to our standard DIA method. This indicates that the small increase in mass range and the resulting higher number of precursors are beneficial justifying the slightly slower cycle time. The achieved high quantitative precision is comparable to that of our standard DIA method.

**Figure 4:**
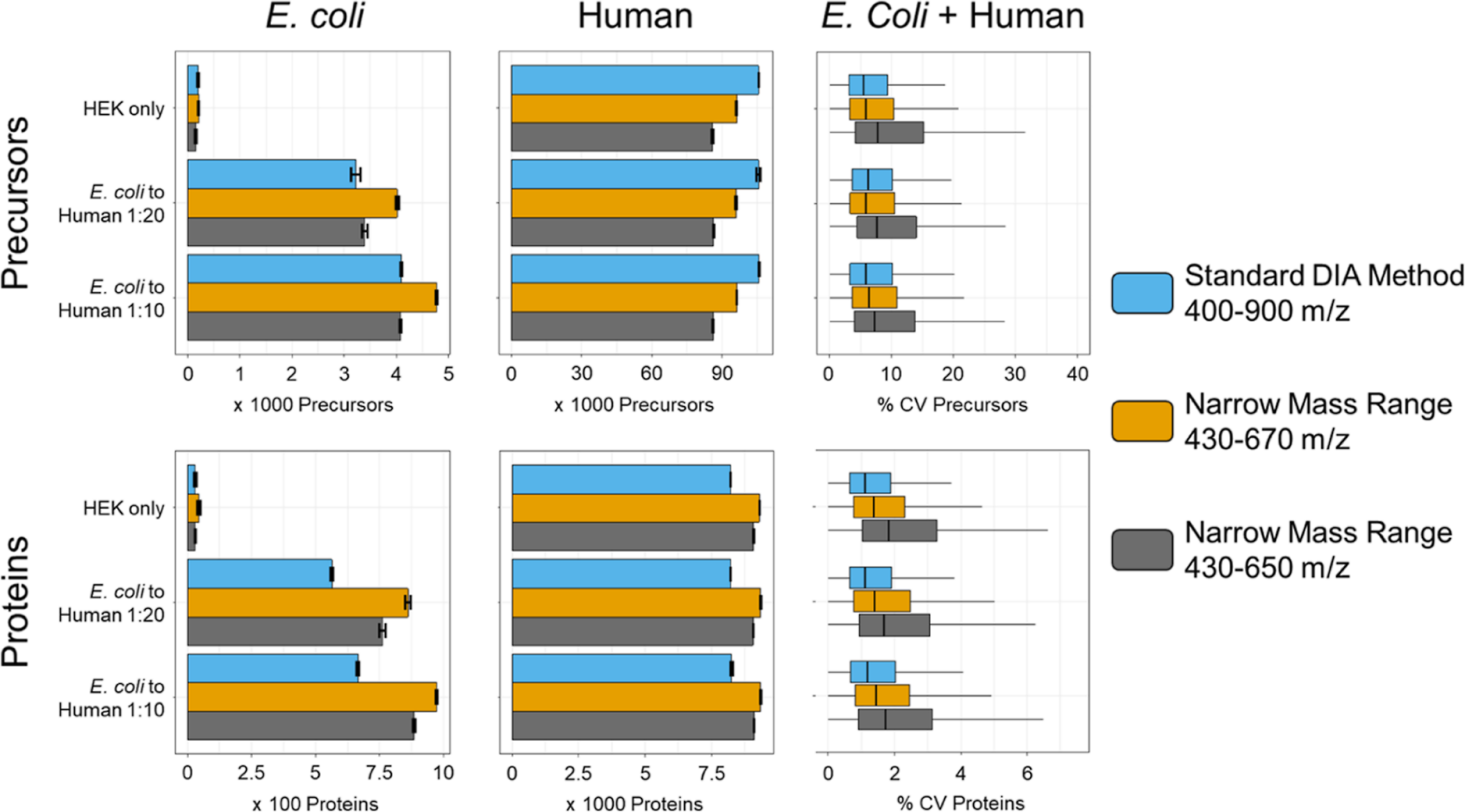
Optimized mass range measurement of HEK *E. coli* dilution. An *E. coli*–HEK dilution series (no *E. coli* = HEK only) was measured in triplicates. DIA-NN was used for data analysis, standard DIA method = 8 *m*/*z* windows +1 *m*/*z* overlap at 15,000 resolution covering 400–900 *m*/*z*. Narrow mass range 430–670 *m*/*z*, 4 *m*/*z* windows (no overlap) at 30,000 resolution covering 430–670 *m*/*z* without using FAIMS. Narrow mass range 430–670 *m*/*z* = same as 430–650 but covering a smaller mass range. Identifications and variance are divided into precursor and protein level, and identifications are additionally divided into species. The barplots show the mean identifications ± the respective standard deviation of identifications within the group.

To further assess the performance of our optimized method, we applied it to the aforementioned HEK *E. coli* dilution series (Figure 4). Notably, we could identify 13.2% more human proteins in the HEK only samples (9293 in total) using our optimized narrow mass range acquisition method compared to an average of 8211 proteins using our standard DIA method. The mean CVs of human proteins found in the HEK-only condition increased only slightly when using our narrow mass range acquisition scheme from 2.7 to 3.2%. However, lowly abundant proteins usually display higher quantitative variances,^42^ raising the question whether the newly identified, extremely lowly abundant proteins are responsible for the observed increase in CVs.

Upon visual inspection, we noticed that we indeed identified more low-abundance proteins using the narrow mass range methods compared to the standard DIA method (Supporting Information Figure S5). When only considering proteins which were identified in all three acquisition methods, we achieved average protein CVs of 3.3, 2.4 and 2.7% percent for the narrow mass range methods 430–650 *m*/*z*, 430–670 *m*/*z* and the standard DIA method, respectively. By using the narrow mass range from 430 to 670 *m*/*z* we could increase protein coverage by 13% and achieve superior quantitative precision as compared to the standard DIA method. We hypothesize that the overall higher protein abundance distribution of the standard DIA method is associated with the higher ratio of observed peptides/proteins, allowing more peptides to be summed up into proteins.

To conclude this part, we confirmed that the original Kawashima method considerably increased proteome coverage over previously published DIA-MS methods and allowed nearly comprehensive coverage of a HEK proteome. However, we identified three major shortcomings of the method including (I) low quantitative precision as compared to our established method, (II) high sample load on column required for deep proteome coverage, and (III) additional complexity of MS setup when employing FAIMS.

We showed that our method achieved much higher quantitative precision, which is essential for sensitive and reliable differential and global proteomics studies. On top, a similar proteome coverage could be obtained without FAIMS in combination with our modified mass range and using much less sample material. However, if preferred, our method is also fully compatible with FAIMS.

### Proteome Analysis of PGC-1α KO/WT Murine Skeletal Muscle

**Figure 5:**
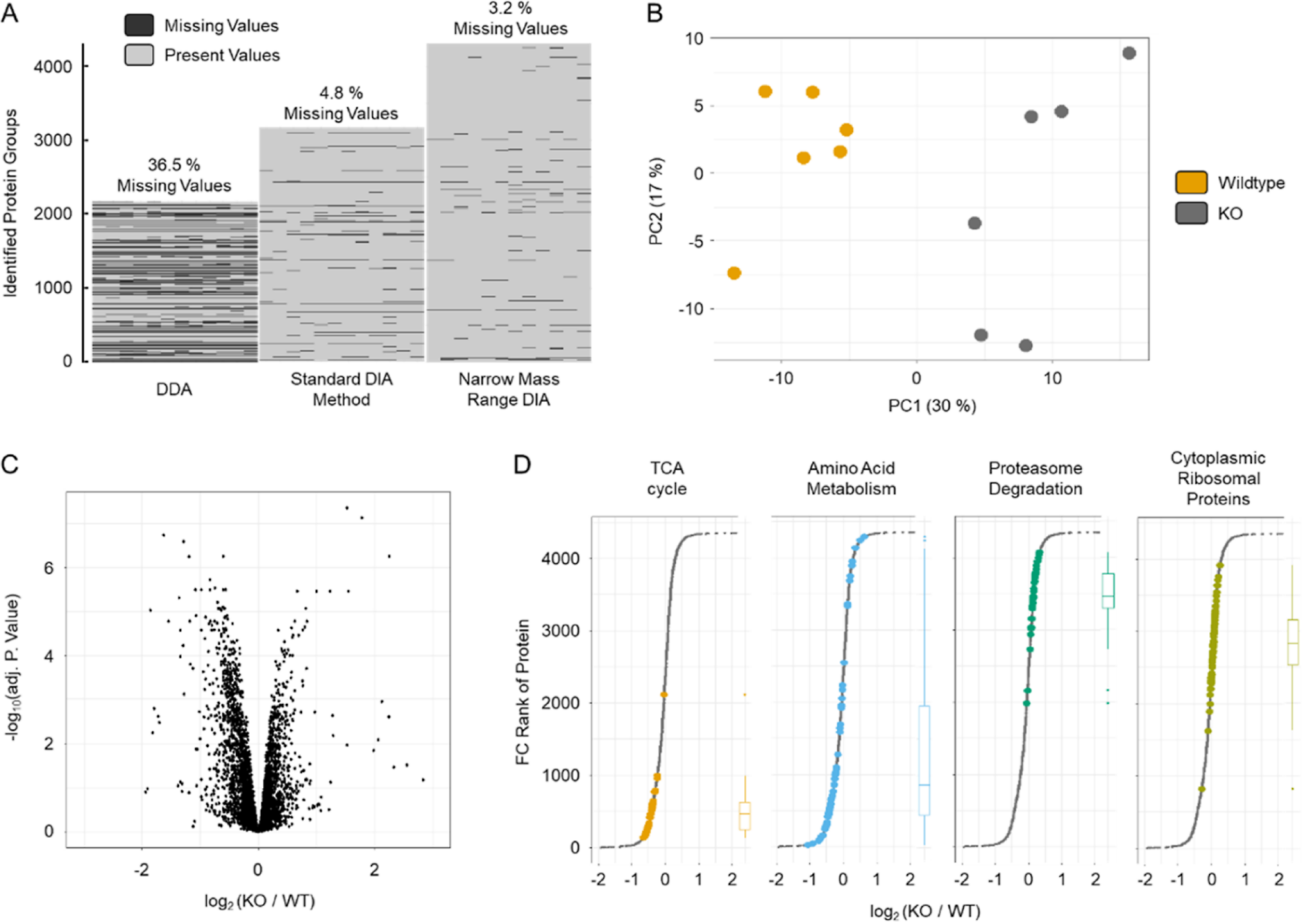
Investigation of mouse muscle knockout. The muscle proteome was compared between mice lacking PGC-1α and WT mice employing DDA, the standard DIA method, and the narrow mass range DIA method. (A) ID numbers of all LC–MS/MS methods and visualization of data completeness. (B) PCA analysis of the comparison using the narrow mass range DIA data. (C) Limma analysis of the comparison using the narrow mass range DIA data. (D) STRING-DB WikiPathWay enrichment “Proteins with Values” using the fold changes of KO/WT. Top = enriched in KO, and bottom = enriched in WT.

To demonstrate the power of our optimized narrow mass range DIA method, we applied it to a system wide proteome analysis of skeletal muscle obtained from WT mice and mice lacking PGC-1α. To highlight the improved proteome coverage of our optimized method, we measured the samples employing a standard DDA and DIA method and the newly developed optimized narrow mass range DIA method. We were able to identify 2179, 3175, and 4307 protein groups using DDA, standard DIA method and narrow mass range DIA, respectively, in the muscle tissue samples analyzed (Figure 5A). To further investigate the validity of our deeper proteome coverage, we compared our DDA, standard DIA and narrow mass range DIA protein group identifications with a previously published comprehensive murine skeletal muscle proteome data set.^23^ Briefly, we downloaded the data, reprocessed it using FragPipe and aligned the identified proteins’ intensities with our three data sets. As expected, owing to the lower coverage, mostly highly abundant proteins were identified with DDA (see Figure S6). Both DIA methods increased the dynamic range covered, with the narrow mass range DIA method identifying the highest number of proteins, including low abundant proteins. Of note, like plasma, skeletal muscle samples have an exceptionally high dynamic protein concentration range that is further enhanced by the presence of the largest protein in the human genome (Titin, 4 MDa) in high amounts.^23,43^ In particular, for bottom-up proteomics LC–MS workflows, after proteolytic cleavage, the high number of generated highly abundant peptides arising from a few very abundant large proteins makes these samples very challenging to analyze extensively. Thus, new LC–MS methods with a higher dynamic range are urgently needed to provide a more holistic view of the muscle proteome to cover relevant adaptations. With our optimized narrow mass range DIA MS approach, we considerably increased the dynamic range over existing MS approaches. This is particularly useful for such high dynamic range samples as it extends coverage to the most populated protein concentration regions (Figure S6). In our skeletal muscle sample analysis, it led to a much higher proteome coverage increase of 36% over our standard DIA method compared to the human cell line analyzed (13%) with its lower dynamic protein concentrations range.

To evaluate whether more relevant proteome alterations could be obtained using the new narrow mass range DIA method, the skeletal muscle samples were analyzed with both DIA methods. As shown in Figure 5B, narrow mass range DIA clearly separated WT from KO in the first component of a Principal Component Analysis (PCA). We then performed limma analyses to obtain fold changes between KO and WT using both the narrow mass range DIA and the standard DIA method data.^28^ This fold change was then used to perform a STRING-DB analysis “Proteins with Values”.^44^ Both methods successfully identified well-described pathways controlled by PGC-1α, such as tricarboxylic acid (TCA) cycle, oxidative phosphorylation, the electron transport chain and fatty acid beta-oxidation that are substantially decreased in muscles lacking PGC-1α (Figures 5D & S7). These findings are in line with previous observations, highlighting the important role of this coactivator in regulating oxidative metabolism.^20,45−47^ Furthermore, proteasome degradation is enriched in the proteome of KO muscles compared to that of WT, which is in accordance with the known role of PGC-1α in protecting muscles from atrophy by suppressing proteasomal degradation.^48^ Strikingly, the novel method was able to detect two additional pathways: TNF-alpha NF-κB Signaling Pathway (enriched in KO) and mRNA Processing (decreased in KO) (Supporting Information Figure S7). Muscle-specific overexpression of PGC-1α has an anti-inflammatory effect in muscle by repressing the action of NF-κB.^49^ In contrast, mice lacking PGC-1α express higher levels of TNFα,^45^ supporting our findings. Moreover, the involvement of PGC-1α in mRNA processing has been demonstrated in vitro.^50−52^ Our results confirm that proteins linked to this process are decreased in the absence of muscle PGC-1α. Therefore, this newly developed method contributes substantially to the detection of a higher number of proteins associated with various pathways and could thereby help identify novel target proteins.

## Conclusions

Here, we present an optimized narrow mass range DIA method capable of identifying up to 9300 protein groups in HEK triplicates. Compared to the original published method introducing the use of narrow mass range 1D-LC-MS DIA, we could reduce the sample amount required to standard levels without compromising proteome coverage. This was achieved by excluding a FAIMS device and optimizing the DIA mass range, thereby making the method accessible to laboratories lacking FAIMS. More importantly, we could show that the quantitative performance of the originally proposed method was considerably lower compared to standard DIA methods, greatly limiting its suitability for quantitative proteome analysis. By systematic evaluation and optimization of the most critical parameters, we could improve the quantitative performance to levels expected from standard DIA methods. Overall, we present a DIA method with extended dynamic range and proteome coverage while preserving the high quantitative precision and accuracy typical of DIA. It is important to note that due to the use of elevated MS resolution and small mass isolation windows, acquisition time was longer than that in our standard DIA method. As this study aimed to provide a deep proteome profiling method with precise and accurate quantification, we did not focus on optimizing sample throughput. However, there is potential to do so if needed. We further demonstrated that the deep coverage is especially suited for analyzing samples with a high dynamic range, as showcased by the identification of additional low-abundance proteins and WikiPathway enrichments from skeletal muscle samples compared with a previously published DIA method. As the method does not require an ion mobility device and is based on optimized DIA-MS parameter settings, it will be applicable and useful to any LC–MS-based proteomics laboratory and facility working with complex and challenging proteome samples.

## Supporting Information Available

Assessment of quantitative accuracy in the spike-in experiment; CVs of the original Kawashima method as published by Kawashima et al.; mass range distribution of precursors in representative DIA experiments without using FAIMS; effect of maximum injection time and resolution on identifications; protein CVs over protein intensities; intensity distribution of identified muscle proteins as compared to a reference data set; STRING-DB analysis: WikiPathway enrichment analysis; number of identified precursors; and number of identified proteins (PDF)
