# Supplementary material for: Robust, Precise, and Deep Proteome Profiling Using a Small Mass Range and Narrow Window Data-Independent-Acquisition Scheme
Source: J Proteome Res. 2024 Jan 26;23(3):1028–38. doi: 10.1021/acs.jproteome.3c00736 (PMC10913089; doi:10.1021/acs.jproteome.3c00736)
Supplement: Supplementary file 1 — pr3c00736_si_001.pdf [file pr3c00736_si_001.pdf]

Supplementary Information

Robust, Precise and Deep Proteome Profiling Using a Small Mass Range and Narrow Window Data-Independent-Acquisition Scheme

‡Klemens Fröhlich [1], ‡Regula Furrer [2], Christian Schori [1], Christoph Handschin [2], Alexander Schmidt [1]*

‡ contributed equally

* corresponding author

1. Proteomics Core Facility, Biozentrum Basel, University of Basel, 4056 Basel, Switzerland
2. Biozentrum Basel, University of Basel, 4056 Basel, Switzerland

Table of Contents:

Supplementary Figure S1. Assessment of quantitative accuracy in spike-in experiment.

Supplementary Figure S2. CVs of the original Kawashima method as published by Kawashima et al.

Supplementary Figure S3. Mass range distribution of precursors in representative DIA experiments without using FAIMS.

Supplementary Figure S4. Effect of Maximum Injection Time and Resolution on Identifications.

Supplementary Figure S5. Protein CVs over protein intensities.

Supplementary Figure S6. Intensity distribution of identified muscle proteins as compared to a reference dataset.

Supplementary Figure S7. STRING-DB analysis: WikiPathway enrichment analysis.

Supplementary Table 1. Number of Identified Precursors as Displayed in Figure 1

Supplementary Table 2. Number of Identified Proteins as displayed in Figure 1

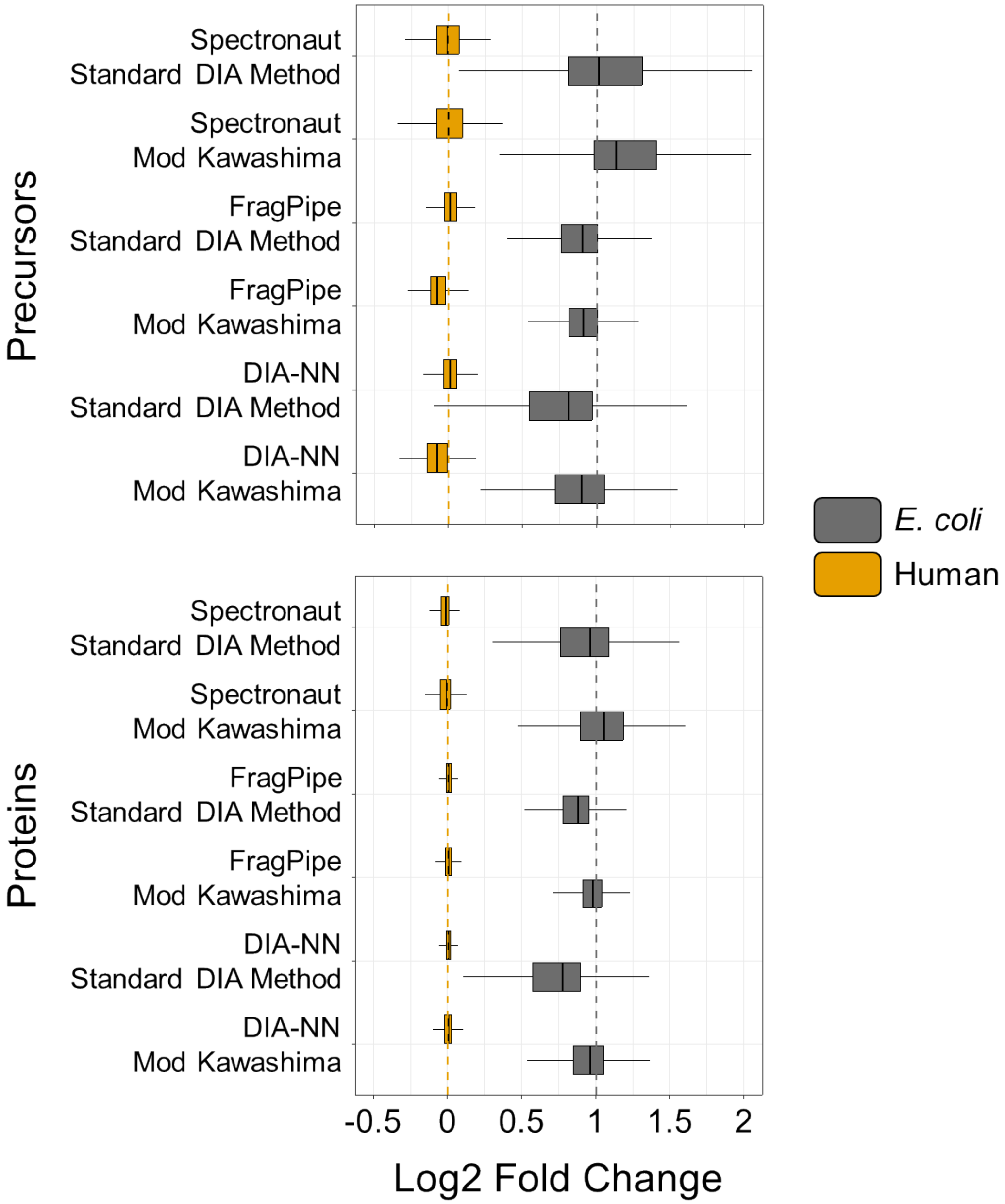

Supplementary Figure S1. Assessment of quantitative accuracy in spike-in experiment. 1:10 vs. 1:20 *E. coli* spike-in dataset was measured using either a modified Kawashima method (4 m/z windows, 500-740 m/z mass range, 30,000 MS2 resolution, staggered window placement, no FAIMS) or the DIA Standard Method (8 m/z windows, 400 - 900 m/z mass range, 15,000 MS2

resolution, no staggering / overlapping window placement, no FAIMS). Dotted lines represent the theoretical fold changes (0 for human, 1 for *E. coli*).

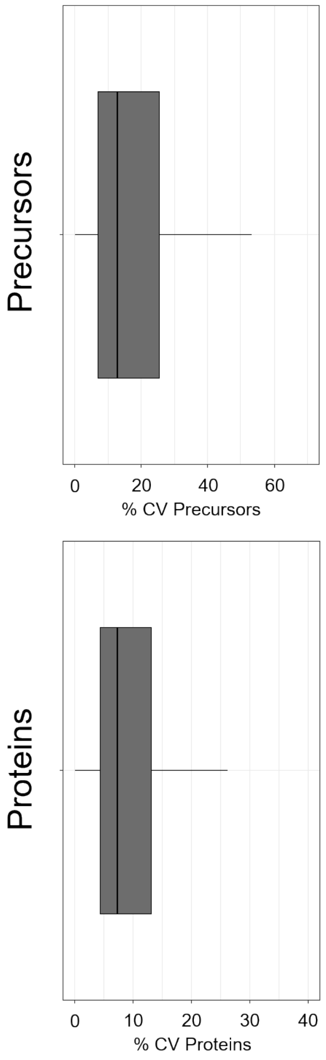

Supplementary Figure S2. CVs of the original Kawashima method as published by Kawashima et al. Original raw data was downloaded and reanalyzed by DIA-NN. The triplicate injection used in

this analysis is the 1000 ng loading suggested by the authors to be used for optimal coverage results.

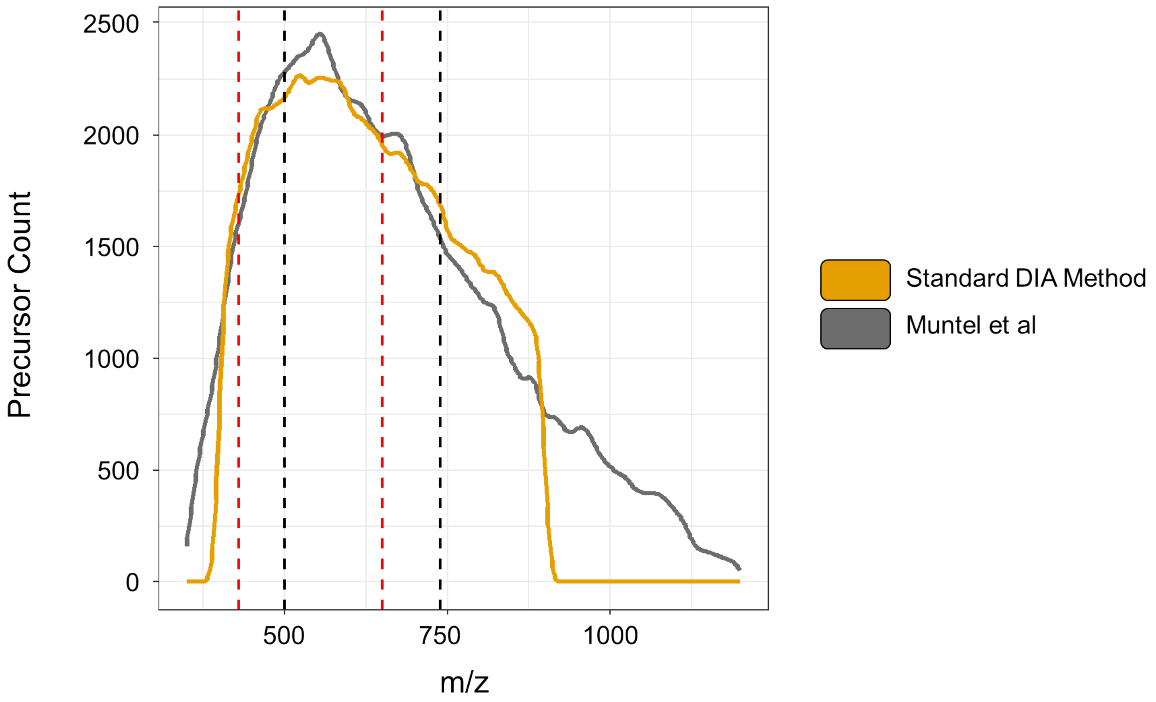

Supplementary Figure S3. Mass range distribution of precursors in representative DIA experiments without using FAIMS. Black dotted lines: Mass Range chosen by Kawashima for an optimized workflow using FAIMS. Red dotted lines: Proposed mass range for maximum number of precursor identification without using FAIMS. Both Standard DIA Method and Muntel et al data have been obtained without FAIMS. Only identifications < 1200 m/z are displayed.

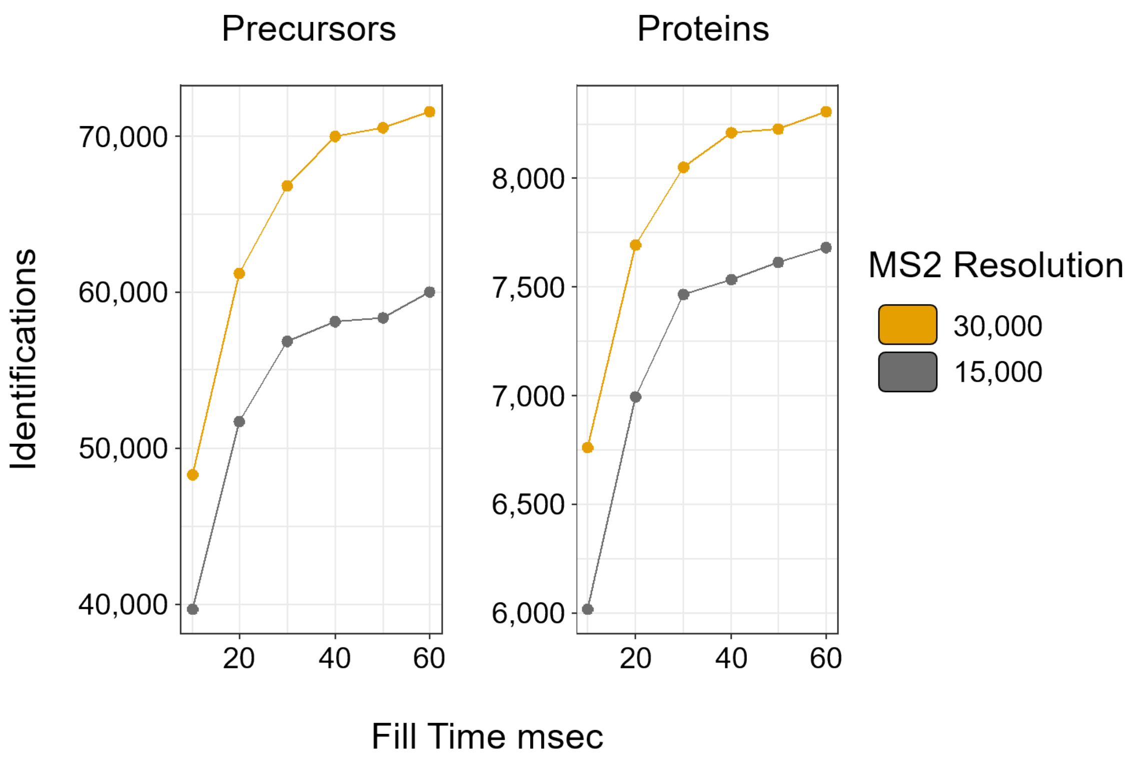

Supplementary Figure S4. Effect of Maximum Injection Time and Resolution on Identifications. HEK lysate was repeatedly injected using different fill times and different fragment spectrum resolution. Data analysis was carried out using DIA-NN without MBR and therefore without library refinement to avoid alignment of identifications from other measurements.

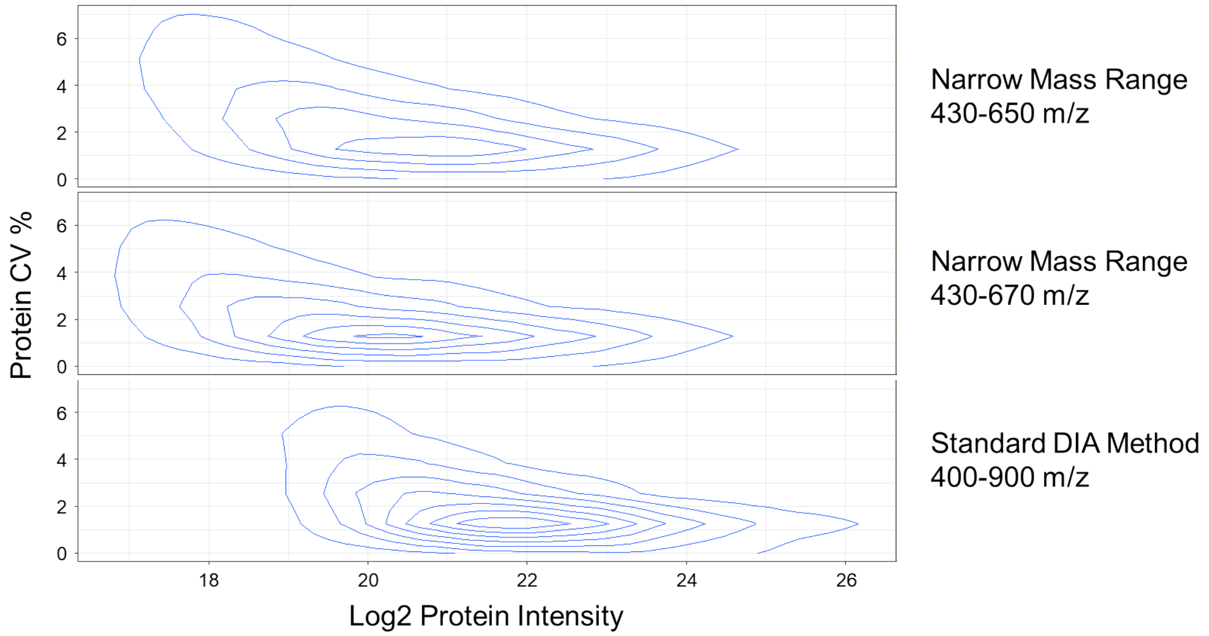

Supplementary Figure S5. Protein CVs over protein intensities. Narrow Mass Range 430 - 650 m/z = 4 m/z windows (no overlap) at 30,000 resolution covering 430 - 650 m/z without using FAIMS. Narrow Mass Range 430 - 670 m/z = same as 430 - 650 but covering a larger mass range. DIA Standard Method = 8 m/z windows + 1 m/z overlap at 15,000 resolution covering 400 - 900 m/z. Protein CVs distribution is depicted over Protein intensity as 2D density map.

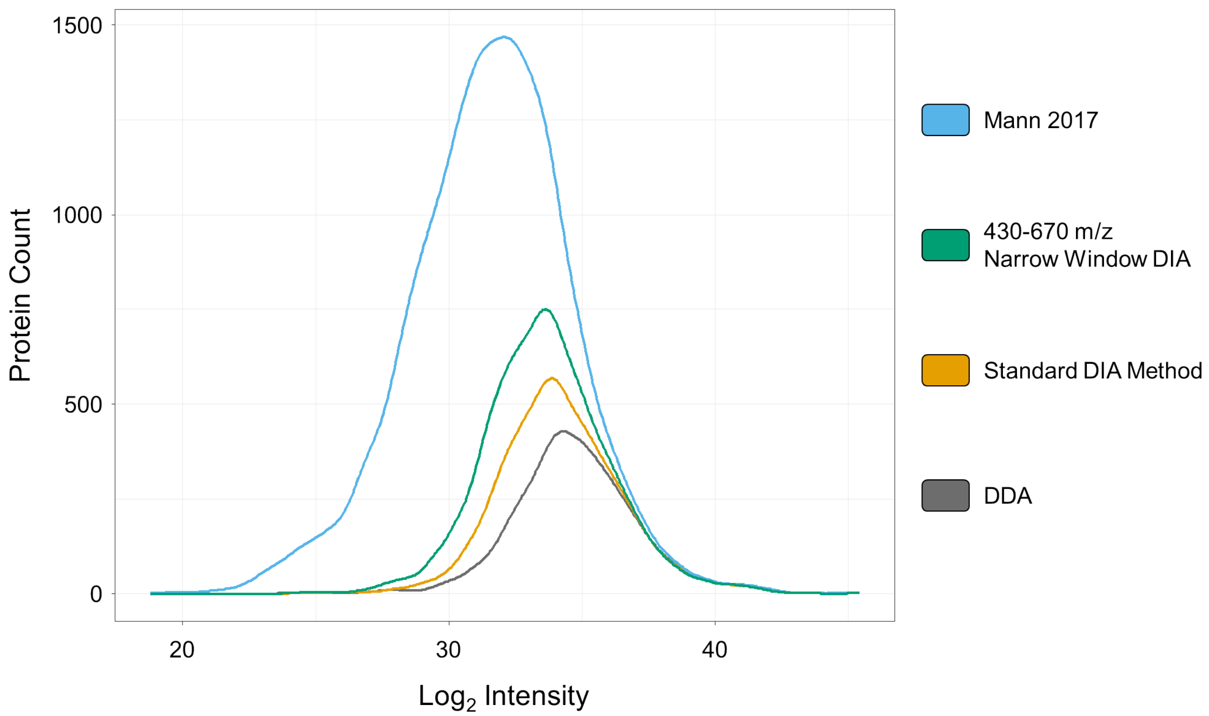

Supplementary Figure S6. Intensity distribution of identified muscle proteins as compared to a reference dataset. Identified proteins of the mouse muscle investigation were compared to a near comprehensive murine muscle dataset. The dataset was reanalyzed using FragPipe and the intensities were copied from the reference dataset to the identified proteins to our own experiments.

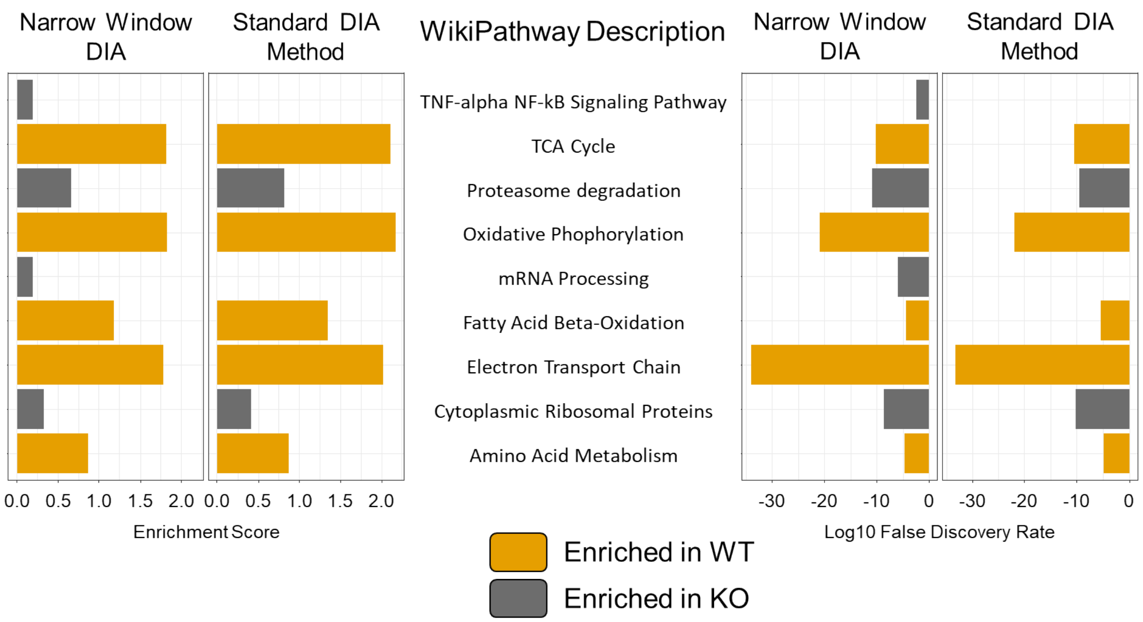

Supplementary Figure S7. STRING-DB analysis: WikiPathway enrichment analysis. Identified WikiPathway activity of the mouse muscle investigation as determined by the DIA Standard Method and the Narrow Mass Range method. The enrichment score of all identified WikiPathways is depicted on the left and the log10(FDR) is depicted on the right. Colors: Top (orange) is enriched in KO, whereas Bottom (gray) is enriched in WT.

Origin	Species	Condition	Method	Software	Mean	SD
DIANN181_Kawash	ECOLI	EC_high	Kawashima	DIA-NN 1.8.1	5095	17
DIANN181_Kawash	ECOLI	EC_low	Kawashima	DIA-NN 1.8.1	4381	19
DIANN181_Kawash	ECOLI	HEK	Kawashima	DIA-NN 1.8.1	288	5
DIANN181_Kawash	HUMAN	EC_high	Kawashima	DIA-NN 1.8.1	94605	252
DIANN181_Kawash	HUMAN	EC_low	Kawashima	DIA-NN 1.8.1	94834	239
DIANN181_Kawash	HUMAN	HEK	Kawashima	DIA-NN 1.8.1	94375	337
DIANN181_Searle	ECOLI	EC_high	Standard	DIA-NN 1.8.1	3687	6
DIANN181_Searle	ECOLI	EC_low	Standard	DIA-NN 1.8.1	2970	44
DIANN181_Searle	ECOLI	HEK	Standard	DIA-NN 1.8.1	185	5
DIANN181_Searle	HUMAN	EC_high	Standard	DIA-NN 1.8.1	97465	302
DIANN181_Searle	HUMAN	EC_low	Standard	DIA-NN 1.8.1	97487	293
DIANN181_Searle	HUMAN	HEK	Standard	DIA-NN 1.8.1	96699	242
FP19_Kawash	ECOLI	EC_high	Kawashima	FragPipe 19	2894	3
FP19_Kawash	ECOLI	EC_low	Kawashima	FragPipe 19	2812	10
FP19_Kawash	ECOLI	HEK	Kawashima	FragPipe 19	88	9
FP19_Kawash	HUMAN	EC_high	Kawashima	FragPipe 19	78791	44
FP19_Kawash	HUMAN	EC_low	Kawashima	FragPipe 19	78792	51
FP19_Kawash	HUMAN	HEK	Kawashima	FragPipe 19	78713	85
FP19_Searle	ECOLI	EC_high	Standard	FragPipe 19	2067	4
FP19_Searle	ECOLI	EC_low	Standard	FragPipe 19	1938	8
FP19_Searle	ECOLI	HEK	Standard	FragPipe 19	68	9
FP19_Searle	HUMAN	EC_high	Standard	FragPipe 19	76695	33
FP19_Searle	HUMAN	EC_low	Standard	FragPipe 19	76724	140
FP19_Searle	HUMAN	HEK	Standard	FragPipe 19	76721	97
SN17_Kawash	ECOLI	EC_high	Kawashima	Spectronaut 17	4409	12
SN17_Kawash	ECOLI	EC_low	Kawashima	Spectronaut 17	4153	33
SN17_Kawash	ECOLI	HEK	Kawashima	Spectronaut 17	267	32
SN17_Kawash	HUMAN	EC_high	Kawashima	Spectronaut 17	99988	472
SN17_Kawash	HUMAN	EC_low	Kawashima	Spectronaut 17	99937	217
SN17_Kawash	HUMAN	HEK	Kawashima	Spectronaut 17	98892	171
SN17_Searle	ECOLI	EC_high	Standard	Spectronaut 17	4077	2
SN17_Searle	ECOLI	EC_low	Standard	Spectronaut 17	3980	11
SN17_Searle	ECOLI	HEK	Standard	Spectronaut 17	377	26
SN17_Searle	HUMAN	EC_high	Standard	Spectronaut 17	121582	29
SN17_Searle	HUMAN	EC_low	Standard	Spectronaut 17	121586	45
SN17_Searle	HUMAN	HEK	Standard	Spectronaut 17	120598	161

Supplementary Table 1. Number of Identified Precursors as Displayed in Figure 1

Origin	Species	Condition	Method	Software	Mean	SD
DIANN181_Kawash	ECOLI	EC_high	Kawashima	DIA-NN 1.8.1	1062	3
DIANN181_Kawash	ECOLI	EC_low	Kawashima	DIA-NN 1.8.1	964	5
DIANN181_Kawash	ECOLI	HEK	Kawashima	DIA-NN 1.8.1	55	4
DIANN181_Kawash	HUMAN	EC_high	Kawashima	DIA-NN 1.8.1	9686	9
DIANN181_Kawash	HUMAN	EC_low	Kawashima	DIA-NN 1.8.1	9707	36
DIANN181_Kawash	HUMAN	HEK	Kawashima	DIA-NN 1.8.1	9705	39
DIANN181_Searle	ECOLI	EC_high	Standard	DIA-NN 1.8.1	640	8
DIANN181_Searle	ECOLI	EC_low	Standard	DIA-NN 1.8.1	559	1
DIANN181_Searle	ECOLI	HEK	Standard	DIA-NN 1.8.1	36	2
DIANN181_Searle	HUMAN	EC_high	Standard	DIA-NN 1.8.1	8074	19
DIANN181_Searle	HUMAN	EC_low	Standard	DIA-NN 1.8.1	8101	14
DIANN181_Searle	HUMAN	HEK	Standard	DIA-NN 1.8.1	8042	21
FP19_Kawash	ECOLI	EC_high	Kawashima	FragPipe 19	728	1
FP19_Kawash	ECOLI	EC_low	Kawashima	FragPipe 19	714	2
FP19_Kawash	ECOLI	HEK	Kawashima	FragPipe 19	45	2
FP19_Kawash	HUMAN	EC_high	Kawashima	FragPipe 19	8610	6
FP19_Kawash	HUMAN	EC_low	Kawashima	FragPipe 19	8614	4
FP19_Kawash	HUMAN	HEK	Kawashima	FragPipe 19	8610	5
FP19_Searle	ECOLI	EC_high	Standard	FragPipe 19	409	1
FP19_Searle	ECOLI	EC_low	Standard	FragPipe 19	397	2
FP19_Searle	ECOLI	HEK	Standard	FragPipe 19	30	4
FP19_Searle	HUMAN	EC_high	Standard	FragPipe 19	6794	2
FP19_Searle	HUMAN	EC_low	Standard	FragPipe 19	6792	3
FP19_Searle	HUMAN	HEK	Standard	FragPipe 19	6788	3
SN17_Kawash	ECOLI	EC_high	Kawashima	Spectronaut 17	855	3
SN17_Kawash	ECOLI	EC_low	Kawashima	Spectronaut 17	819	7
SN17_Kawash	ECOLI	HEK	Kawashima	Spectronaut 17	132	16
SN17_Kawash	HUMAN	EC_high	Kawashima	Spectronaut 17	9142	25
SN17_Kawash	HUMAN	EC_low	Kawashima	Spectronaut 17	9144	8
SN17_Kawash	HUMAN	HEK	Kawashima	Spectronaut 17	9107	6
SN17_Searle	ECOLI	EC_high	Standard	Spectronaut 17	578	1
SN17_Searle	ECOLI	EC_low	Standard	Spectronaut 17	555	5
SN17_Searle	ECOLI	HEK	Standard	Spectronaut 17	105	13
SN17_Searle	HUMAN	EC_high	Standard	Spectronaut 17	7831	6
SN17_Searle	HUMAN	EC_low	Standard	Spectronaut 17	7835	4
SN17_Searle	HUMAN	HEK	Standard	Spectronaut 17	7815	5

Supplementary Table 2. Number of Identified Proteins as displayed in Figure 1
